# Mild Parkinsonian Signs Are Related to Lower Cortico-Striatal Connectivity in Executive Networks

**DOI:** 10.1093/geroni/igab046.615

**Published:** 2021-12-17

**Authors:** James Hengenius, Theodore Huppert, Andrea Rosso, Caterina Rosano

**Affiliations:** University of Pittsburgh, Pittsburgh, Pennsylvania, United States

## Abstract

Mild Parkinsonian signs (MPS) affect up to 24% of community-dwelling older adults. We hypothesize that MPS are associated with Parkinson’s-like alterations of functional connectivity (FC) in sensorimotor, executive, and reward cortico-striatal networks. Participants (N=266; mean age=83; 57% female) without Parkinson’s completed resting-state fMRI and Unified Parkinsonian Disease Rating Scale (UPDRS). FC between striatum and cortex was measured within each network. Logistic regression tested associations of each network’s FC with MPS (UPDRS>0), adjusted for MPS risk factors, then including white matter hyperintensities (WMH). MPS was associated with lower cortical-striatal FC in the left executive cortico-striatal network (OR [95%CI]: 0.188 [0.043,0.824]). Association survived adjusting for risk factors (0.162 [0.030,0.874]) but was attenuated after including WMH (0.209 [0.036, 1.200]). In stratified analyses, left executive cortico-striatal FC was associated with MPS only for those with higher WMH (0.077 [0.010,0.599]). Future work should examine whether higher FC protects against the influence of WMH on MPS.

